# Malignancy of Cancers and Synthetic Lethal Interactions Associated With Mutations of Cancer Driver Genes

**DOI:** 10.1097/MD.0000000000002697

**Published:** 2016-03-03

**Authors:** Xiaosheng Wang, Yue Zhang, Ze-Guang Han, Kun-Yan He

**Affiliations:** From the School of Basic Medicine and Clinic Pharmacy (XW), China Pharmaceutical University, Nanjing; The First Clinical College of Harbin Medical University (YZ), Harbin, China; Division of Genetics and Development (YZ), The Toronto Western Research Institute, Toronto Western Hospital, University Health Network, Toronto, Ontario, Canada; and Key Laboratory of Systems Biomedicine (Ministry of Education) (Z-GH, K-YH), Shanghai Center for Systems Biomedicine, Shanghai Jiao Tong University, Shanghai, China.

## Abstract

The mutation status of cancer driver genes may correlate with different degrees of malignancy of cancers. The doubling time and multidrug resistance are 2 phenotypes that reflect the degree of malignancy of cancer cells. Because most of cancer driver genes are hard to target, identification of their synthetic lethal partners might be a viable approach to treatment of the cancers with the relevant mutations.

The genome-wide screening for synthetic lethal partners is costly and labor intensive. Thus, a computational approach facilitating identification of candidate genes for a focus synthetic lethal RNAi screening will accelerate novel anticancer drug discovery.

We used several publicly available cancer cell lines and tumor tissue genomic data in this study.

We compared the doubling time and multidrug resistance between the NCI-60 cell lines with mutations in some cancer driver genes and those without the mutations. We identified some candidate synthetic lethal genes to the cancer driver genes *APC*, *KRAS*, *BRAF*, *PIK3CA*, and *TP53* by comparison of their gene phenotype values in cancer cell lines with the relevant mutations and wild-type background. Further, we experimentally validated some of the synthetic lethal relationships we predicted.

We reported that mutations in some cancer driver genes mutations in some cancer driver genes such as *APC*, *KRAS*, or *PIK3CA* might correlate with cancer proliferation or drug resistance. We identified 40, 21, 5, 43, and 18 potential synthetic lethal genes to *APC*, *KRAS*, *BRAF*, *PIK3CA*, and *TP53*, respectively. We found that some of the potential synthetic lethal genes show significantly higher expression in the cancers with mutations of their synthetic lethal partners and the wild-type counterparts. Further, our experiments confirmed several synthetic lethal relationships that are novel findings by our methods.

We experimentally validated a part of the synthetic lethal relationships we predicted. We plan to perform further experiments to validate the other synthetic lethal relationships predicted by this study.

Our computational methods achieve to identify candidate synthetic lethal partners to cancer driver genes for further experimental screening with multiple lines of evidences, and therefore contribute to development of anticancer drugs.

## Abstract 2

Supplemental Digital Content is available in the text

## INTRODUCTION

Numerous studies have shown that gene mutations underlie the development of all types of cancers.^1^ Gene mutations occur in 2 ways: inherited from a parent (germline mutations) or acquired during a personal lifetime (somatic mutations). It was estimated that approximately 90% of cancer genes show somatic mutations and 20% show germline mutations.^1^ To investigate gene mutations in cancers and develop targeted anticancer drugs, human cancer cell lines are being widely used.^2^ For example, the National Cancer Institute's NCI-60 cell lines have been extensively characterized and frequently used as a screening tool for discovery of anticancer drugs.^3^ Based on the panel of NCI-60 cell lines, the frequently-mutant genes in cancers have been identified, such as *APC*, *BRAF*, *CDKN2A*, *KRAS*, *PIK3CA*, *PTEN*, *TP53*, etc.^3,4^ For the NCI-60 cell lines, the phenotypes such as doubling time (the period of time required for cell lines to double in size) and multidrug resistance (MDR) may indicate the degree of malignancy.^5–8^ With the gene mutation and cancer cell phenotypes information, an important question arises: to what degree the mutations of some genes correlate with high malignancy of cancers? In the present study, we initially investigate the correlation between gene mutations and cancers’ malignancy based on the gene mutation and malignancy phenotypes (doubling time and MDR) data for the NCI60 cell lines since so far such investigation is lacking.

RNAi screening is an approach that facilitates the systematic assessment of the effect of gene deregulation on cell phenotypes such as cell death.^9^ The RNAi screening technology can be used for identification of synthetic lethal partners. Two genes are synthetic lethal if dis-regulation of either alone does not result in cell death but dis-regulation of both leads to death of cells.^10^ Thus, abrogation of gene *X* that is synthetic lethal to gene *Y* should selectively kill *Y*-mutant cells and spare the cells without *Y* mutations. The synthetic lethality concept has been used for discovery of anticancer drugs, which may target some genes whose synthetic lethal partners are frequently mutated in cancers but are hardly druggable such as the tumor suppressor genes *APC* and *TP53*, or have severe drug resistance such as the oncogene *KRAS* and *BRAF*.^11,12^ The standard approach to systematical identification of synthetic lethal genes is based on genome-wide or kinome-wide RNAi screening.^13^ However, large-scale synthetic lethal RNAi screening strategy is laborious and time consuming. Its efficiency could be improved by first identifying differentially expressed genes between isogenically paired cell lines (hereafter refer to single gene mutant versus wild-type), and then performing a focused RNAi screening on the differentially expressed genes to examine their synthetic lethality to the mutant gene.^7,8,12^ Wang and Simon^12^ proposed a method to computationally prescreen synthetic lethal genes to *p53* using gene expression profiles. They identified 98 kinase genes that are potential therapeutic targets for *p53*-mutant cancers. A limitation of that study is that the candidate synthetic lethal genes to *p53* identified may harbor many false positives because their underlying presumption is not necessarily true that the gene expression difference is a result of altered gene mutation status.

RNAi screening uses a short interfering RNA (siRNA) to suppress expression of specific genes. The degree of suppression of the targeted gene is often highly variable due to on-target and off-target effects of siRNA.^9^ In (9) the authors proposed a computational method to quantify gene-specific suppression phenotype. They generate a per-gene value for each sample—gene phenotype value (GPV), quantifying the suppression effect for a specific gene in an individual cell line by siRNA reagents. Furthermore, the authors affirmed that the GPV reflects the degree of dependency of an individual cell line's viability on a specific gene, with a lower GPV representing high viability dependency of a cell line on the gene. If we perform the 2-class comparison between a group of cell lines with mutations of some gene and another group of cell lines without mutations of the gene, we could identify the genes with significantly lower GPVs in the mutant cell lines than in the wide-type cell lines. It means that the mutant cell lines have higher viability dependency on the identified genes than the wide-type cell lines. In the other words, the identified genes could be synthetic lethal to the mutant gene. On the basis of the approach, we identified the potential synthetic lethal genes to *APC*, *KRAS*, *BRAF*, *PIK3CA*, and *TP53*, respectively. Then we performed an examination of the literature to evaluate other evidence for the putative synthetic lethality relationships. Furthermore, we compared expression of the identified genes in between mutant cancer cells/tumor samples and wide type, and identified a portion of genes that show the significantly higher expression level in the mutant cancer cells/tumor samples. In addition, we examined the drug sensitivity differences between NCI-60 cell lines with gene mutations and NCI-60 cell lines without the mutations for the compounds that target the kinases encoded by the genes among the potential synthetic lethal genes identified. Finally, we performed experiments to validate some of the synthetic lethality relationships we computationally identified.

## METHODS

### Datesets

We obtained the NCI-60 cell lines’ phenotype data (doubling time and MDR) from the CellMiner database,^6,14^ and the gene mutation data of NCI-60 cancer cell lines from the publication^4^ (http://mct.aacrjournals.org/content/5/11/2606/T3.expansion.html). The GPVs for the 102 Achilles cancer cell lines are from the publication,^9^ whereas the gene mutation information for the 102 Achilles cancer cell lines is from the Cancer Cell Line Encyclopedia (CCLE) project.^2^ The microarray gene expression dataset for the 102 Achilles cancer cell lines is also from the CCLE project. The microarray gene expression dataset for the NCI-60 cell lines (Affymetrix U95A data from Novartis) is downloaded from the Developmental Therapeutics Program NCI/NIH website https://wiki.nci.nih.gov/display/NCIDTPdata/Molecular+Target+Data. We downloaded the RNA-Seq gene expression dataset for glioblastoma multiforme (GBM) and the microarray gene expression dataset for breast invasive carcinoma (BRCA) from the TCGA website https://tcga-data.nci.nih.gov/tcga/. All of the gene expression datasets were used for the gene differential expression analysis. Ethical approval was waived since we used only publicly available data and materials in this study.

### Comparisons of Doubling Time and MDR Between Mutant and Wide-Type Cell Lines

We compared doubling time and MDR between the mutant NCI-60 cell lines and the wide-type NCI-60 cell lines using *t*-test statistics (1-sided, the hypothesis of less doubling time and stronger MDR in the mutant cell lines). We performed the class comparisons based on the mutant status of NCI-60 cell lines for genes *APC*, *BRAF*, *CDKN2A*, *KRAS*, *PIK3CA*, and *PTEN*, respectively. A detailed description of mutation status of these genes in each NCI-60 cell line is shown in the supplementary Table S1. The numbers of the mutant and wide-type NCI-60 cell lines used for the class comparisons are given in the supplementary Table S2.

### Identification of Potential Synthetic Lethal Genes to APC, KRAS, BRAF, PIK3CA, and TP53

We first identified the genes with differential GPVs between the mutant cell lines and the wild-type cell lines among 102 Achilles cancer cell lines using the univariate *t*-test at a 2-sided significance level of 0.001. We also performed univariate permutation tests with 10,000 permutations of the class label (mutant or wide-type) to measure the significance of individual genes. The proportion of the permutations that gave a *t*-test *P* value as small as obtained with the true class labels was the univariate permutation *P* value for that gene. To adjust for multiple tests, we reported the false discovery rate (FDR) for each gene identified. The FDR was estimated using the method of Benjami and Hochberg.^15^ This procedure was implemented with the class comparison between groups of arrays tools in BRB-ArrayTools.^16^ We selected the genes that showed significantly lower relative GPVs in the mutant cell lines as the potential synthetic lethal genes to the mutant genes. This procedure was carried out for *APC*, *KRAS*, *BRAF*, *PIK3CA*, and *TP53* mutation status in cell lines, respectively (we did not include *CDKN2A* and *PTEN* in the analysis because few cell lines have mutations of them in this dataset). A detailed description of mutation status of these genes in each Achilles cell line is shown in the supplementary Table S1. The numbers of the mutant and wide-type Achilles cancer cell lines used for the class comparisons are given in the supplementary Table S3.

### Comparisons of Expression of the Potential Synthetic Lethal Genes in Mutant and Wide-Type Cancers

For the potential synthetic lethal genes identified, we compared their expression in between mutant and wide-type cell lines or tumors (Achilles cell lines, NCI-60 cell lines, and TCGA (the Cancer Genome Atlas) tumor samples, respectively) using *t* test. The numbers of samples in each class for Achilles cell lines and NCI-60 cell lines are shown in the supplementary Table S3 and Table S2, respectively. The numbers of samples in each class for the TCGA tumors are summarized in the supplementary Table S4. The significantly more highly expressed genes in mutant samples than in wide-type were identified (*P* value <0.05, fold change ≥1.2) and further analyzed.

### Comparison of Drug Sensitivity Between 2 Groups of Cell Lines

We compared drug sensitivity (GI50) between the mutant NCI-60 cell lines and the wild-type NCI-60 cell lines using *t*-test statistics (1-sided, the hypothesis of higher sensitivity in the mutant cell lines). GI50 is the concentration required to inhibit growth of cancer cell lines by 50%. The lower GI50 value means higher drug sensitivity.

### Cells and Reagents

Human cells from colorectal adenocarcinoma, LoVo, SW480, and SW620 were maintained in RPMI 1640 supplemented with 10% fetal bovine serum (FBS), 100 U/mL penicillin, and 0.1 mg/mL streptomycin. The human cells from glioblastoma, TP366, and LN229 cells, and hepatocellular carcinoma, HepG2, Hep3B, and Huh7 were maintained in DMEM with 10% FBS, 100 U/mL penicillin, and 0.1 mg/mL streptomycin. All the cells were kept in a humidified incubator at 37°C and a 5% CO_2_ atmosphere. The PD-0332991 and 68C091 were from Sigma (St. Louis, MD).

### In Vitro Proliferation Assay

Experimental procedures for in vitro proliferation assay are as described in (5). Briefly, 3×10^3^ (for MTT assay) or 1 × 10^3^ (for CCK8 assay) cells were seeded in a 96-well plate in quadruplicate or in hepatuplicate. After 24 hours, different concentrations of drugs or vehicle were added with fresh medium. Cells were incubated at 37°C for 3 days followed by an MTT assay or as indicated time points followed by CCK8 assay. The experiments were repeated at least twice.

### siRNA Knockdown

The scrambled siRNA and target-specific sequences synthesized by GenePharma (Shanghai, China) were as follows: siTDO2, 5’-AGU GAU AGG UAC AAG GUA UUU-3’; siCTNNB1#1, 5’-UUG UUA UCA GAG GAC UAA AUA-3’; siCTNNB1#2, 5’-UCU AAC CUC ACU UGC AAU AAU-3’; siCSNK1A1#1, 5’-GCA AGC UCU AUA AGA UUC UUC-3’; siCSNK1A#2, 5’-GCA GAA UUU GCG AUG UAC UUA-3’. Cells were transfected with siRNA using Lipofectamine 2000 (Invitrogen, Boston, MA) according to the manufacturer's instruction. After 36 hours, the transfected cells were split into 6-well plates for knockdown confirmation and 96-well plates for cell proliferation examination.

### RNA Extraction and RT-PCR

Total RNA was isolated from various cell lines using the TRIzol reagent (Invitrogen, Boston, MA). Two micrograms of RNA was transcribed into complementary DNA by PrimeScript (TaKaRa) in 20 μL, and 0.2 μL was subjected to RT-PCR. The condition for PCR was 25 cycles (except 35 cycles for *TDO2*) of denaturation (94°C/15 s), annealing (55°C/15 s), and extension (72°C/30 s), and 1 cycle of final extension (72°C/5 min). Glyceraldehyde-3-phosphate dehydrogenase (*GAPDH*) was used as an internal control. The amplified PCR products were separated by electrophoresis on a 2.5% agar gel containing ethidium bromide. PCR primers (*TDO2* forward, 5′-AAGGTTGTTTCTCGGATGCAC-3′; *TDO2* reverse, 5′-TGTCATCGTCTCCAGAATGGAA-3′; *CSNK1A1* forward, 5′-AGTGGCAGTGAAGCTAGAATCT-3′; *CSNK1A1* reverse, 5′-CGCCCAATACCCATTAGGAAGTT-3′; *CTNNB1* forward, 5′-CATCTACACAGTTTGATGCTGCT-3′; *CTNNB1* reverse, 5′-GCAGTTTTGTCAGTTCAGGGA-3′; *GAPDH* forward, 5′-GAAGGTGAAGGTCGGAGTC-3′; *GAPDH* reverse, 5′-GAAGATGGTGATGGGATTTC-3′) were synthesized by Sangon (Shanghai, China).

## RESULTS

### Identification of the Cancer Driver Genes Whose Mutations May Correlate With Proliferation or Drug Resistance of Cancers

We compared doubling time and MDR between the mutant and wild-type NCI-60 cell lines and found that the mutant cell lines have less doubling time or stronger MDR than the wild-type cell lines in terms of several cancer driver genes’ mutations (Table 1). For example, the *APC* or *KRAS* mutant cell lines show less doubling time than the wild-type (*P* value <0.05), and the *PIK3CA* mutant cell lines show stronger MDR than the wild-type (*P* value = 0.034). Moreover, for mutations of *APC*, *KRAS*, and *PIK3CA*, the less doubling time may likely correlate with stronger MDR (Table 1). In fact, the correlation coefficient (Pearson) between doubling time and MDR is −0.09 for all the cell lines, but −0.27, −0.27, and −0.25 for *APC*, *KRAS*, and *PIK3CA* mutant cell lines, respectively.

**TABLE 1:**
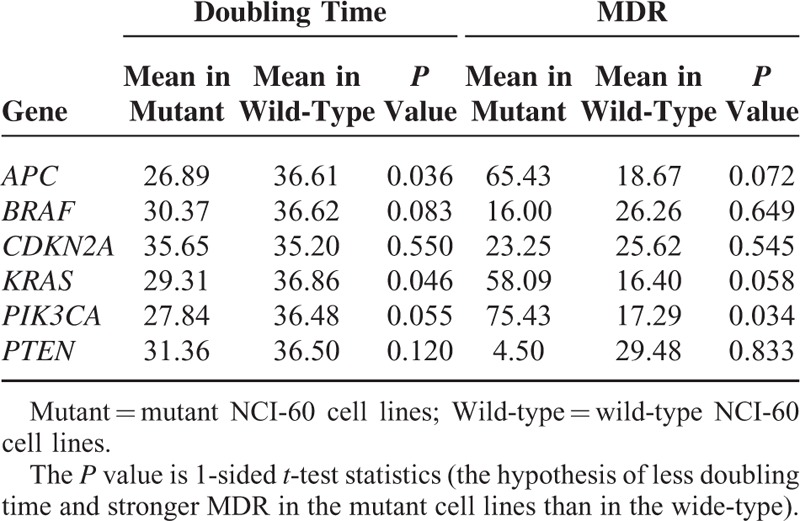
Comparisons of Doubling Time and MDR Between Mutant and Wide-Type Cell Lines

As less doubling time implies faster proliferation rate, the mutation of *APC* or *KRAS* may lead to enhanced malignancy of cancers. On the other hand, as stronger MDR implies severer drug resistance of cancer cell lines, the mutation of *PIK3CA* may confer stronger drug resistance. This is in line with a recent study that revealed that *PIK3CA* mutations may confer resistance in Her2-positive breast cancer.^17^

### Identification of Potential Synthetic Lethal Genes to APC, KRAS, BRAF, PIK3CA, and TP53

To develop rational novel drugs targeting the cancers with mutations of the cancer diver genes, we applied the computational approach to identify 40, 21, 5, 43, and 18 potential synthetic lethal genes to *APC*, *KRAS*, *BRAF*, *PIK3CA,* and *TP53*, respectively. Table 2 lists all of these genes. The results of statistical tests including parametric *P* value, FDR, and permutation *P* value for each gene identified are shown in the supplementary Table S5.

**TABLE 2:**
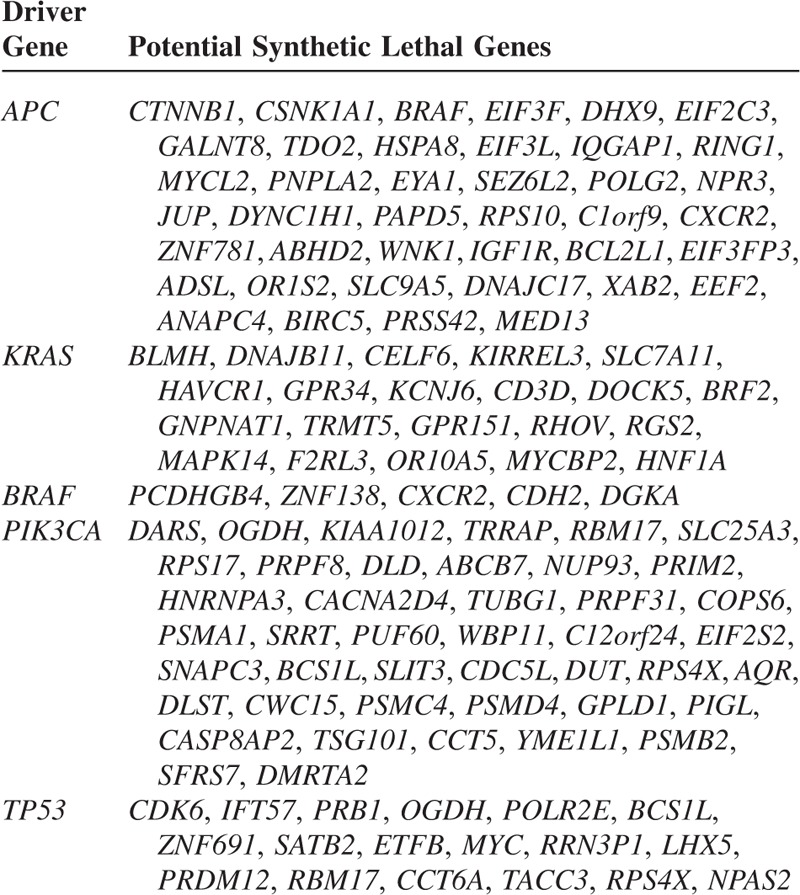
Potential Synthetic Lethal Genes Identified

Interestingly, our results include many synthetic lethal interactions that have been proved by previous studies. For example, *JUP*^18^ and *IGF1R*^19^ have been experimentally proved to interact with *APC*. *IGF1R* has been shown to interact with *APC* indirectly via regulation of the E-cadherin/b-catenin complex.^19^*DOCK5*, *RGS2*, and *F2RL3* have been verified to be synthetic lethal to *KRAS* by Lou et al through a genome-wide RNAi screening.^20^*KCNJ6* is another synthetic lethal gene to *KRAS* as identified in^21^.

Among the potential synthetic lethal genes to *TP53* we identified (Table 2), *TACC3* was found to be synthetic lethal to *TP53* by Krastev et al's standard RNAi screening.^22^ Another important candidate of synthetic lethal genes to *TP53* we identified is the oncogene *MYC*. *MYC* codes for a transcription factor that regulates the expression of many genes. Its mutations have been found in many cancers.^23^ Many studies have shown that p53 can repress *MYC*'s expression and *TP53* mutant cancers may depend on upregulation of *MYC*.^24,25^ These evidences support our inference that there may exist a synthetic lethality relationship between *TP53* and *MYC*.

### Identification of Highly Expressed Genes in Mutant Cancer Cell Lines Among the Potential Synthetic Lethal Genes

It would be not unexpected that the viability of cancer cells likely depends on hyperactivation of oncogenes. Thus, we speculate that the highly expressed genes in cancer cells with gene mutations could be synthetic lethal to the mutant genes. There are a portion of potential synthetic lethal genes identified showing significantly higher expression in the mutant Achilles cell lines than in the wide-type (*P* value <0.05). These genes include *DOCK5*, *PIGL*, *CTNNB1*, *GALNT8*, *TDO2*, *ABHD2*, *DNAJC17*, *ANAPC4*, and *OGDH* (Table 3), which might be potential therapeutic targets for the cancers with related mutations. For example, the gene *CTNNB1* has more than 1.5-fold higher expression in the *APC* mutant cell lines than in the *APC* wide-type cell lines, raising the possibility that the viability of *APC* mutant cancer cells might depend on *CTNNB1*'s expression. However, together with further experimental validation, we may conclude that anticancer drugs that inhibit the expression of *CTNNB1* might be useful in treating *APC* mutant cancers. Strikingly, *TDO2* has more than 3-fold higher expression in the *APC* mutant cell lines than in the *APC* wide-type cell lines, indicative of the possibility of high dependence of *APC* mutant cancer cells on its expression.

**TABLE 3:**
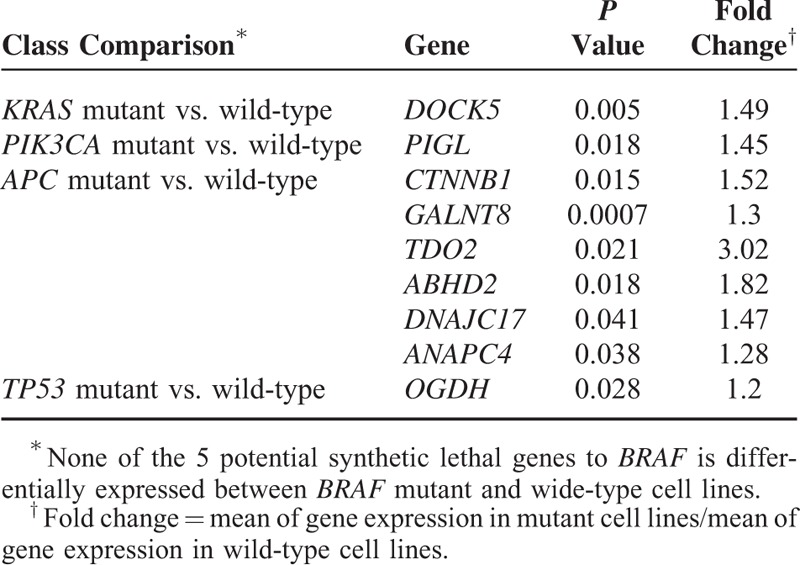
Highly Activated Genes in Mutant Achilles Cell Lines

We also identified several genes that show significantly higher expression in the mutant NCI-60 cell lines than in the wide-type (*P* value <0.05). These genes include *BLMH*, *ABHD2*, *BCL2L1*, and *JUP* (Table 4). These genes encode proteins that are involved in oncogenesis or have unknown function.^26,27^ Among them, *ABHD2* is also highly expressed in the *APC* mutant Achilles cell lines. This gene encodes a protein containing a catalytic domain found in a wide range of enzymes. The function of this protein has not been determined. Our results suggest that this gene could be synthetic lethal to *APC* since it has lower GPVs, and is highly expressed in the *APC* mutant cancer cell lines. *JUP* encodes a cytoplasmic protein that belongs to the catenin family whose members actively interact with *APC*.^28^*BLMH* encodes a cytoplasmic cysteine peptidase that metabolically inactivates the glycopeptide bleomycin, an essential component of combination chemotherapy regimens for cancer.^29^ Our results suggest that this gene could be synthetic lethal to *KRAS* and is a potential therapeutic target for *KRAS* mutant cancers.

**TABLE 4:**
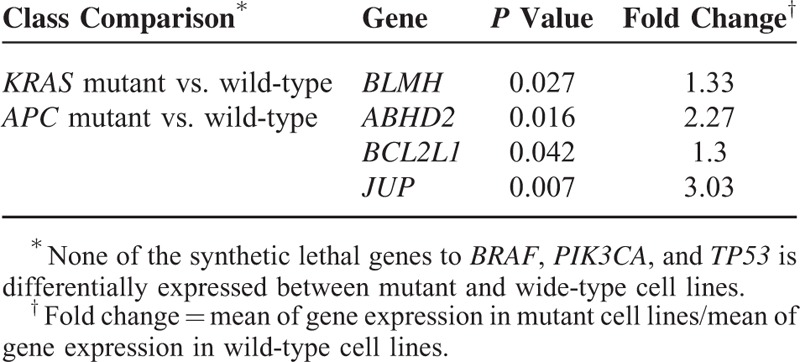
Highly Activated Genes in Mutant NCI-60 Cell Lines

### Identification of Highly Expressed Genes in Mutant Tumors Among the Potential Synthetic Lethal Genes

We also compared the expression of the synthetic lethal genes identified in between mutant and wide-type tumor samples from TCGA. Due to a small proportion of samples with *KRAS*, *BRAF*, *PIK3CA*, and *APC* mutations annotated in the GBM dataset, we only compared the gene expression difference between *TP53* mutant and wide-type GBMs. Similarly, due to a small proportion of samples with *KRAS*, *BRAF*, and *APC* mutations annotated in the BRCA dataset, we compared the gene expression difference between *TP53* mutant and wide-type BRCAs, and between *PIK3CA* mutant and wide-type BRCAs. Table 5 presents the significantly more highly expressed genes in the mutant tumors than in the wide-type (*P* value <0.05).

**TABLE 5:**
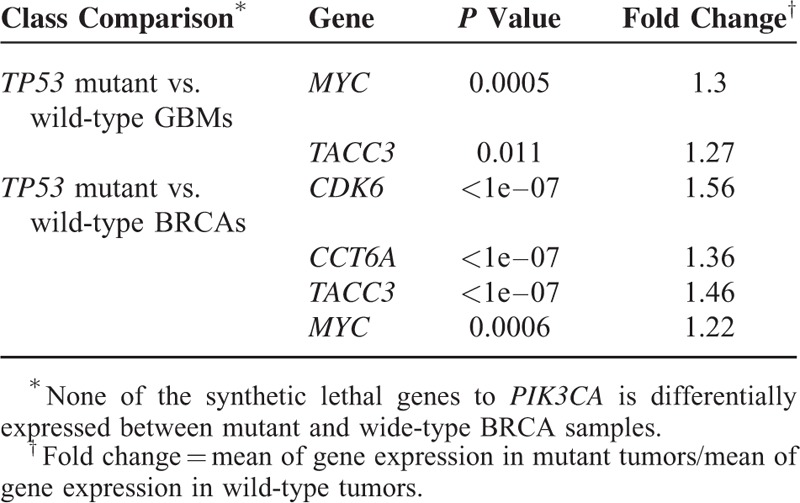
Highly Activated Genes in Mutant Tumors

Table 5 shows that *TACC3* and *MYC* are much more highly expressed in *TP53* mutant GBMs and *TP53* mutant BRCAs compared with respective wide-types. This result indicates that *TP53* mutant cancer cells could rely on the high expression of *TACC3* or *MYC* for survival, predicting the synthetic lethality relationship between *TACC3* and *TP53*, and between *MYC* and *TP53*. The other highly expressed genes in *TP53* mutant BRCAs include *CDK6* and *CCT6A* which are likely to be synthetic lethal to *TP53* (Table 5). Among them, *CDK6* encodes a member of the cyclin-dependent protein kinase (*CDK*) family. This gene has been found to be upregulated in several types of cancers.^30,31^

### Identification of the Potential Synthetic Lethal Genes That Encode Kinases

Discovery of cancer-related kinases and development of kinase inhibitors have been an active research field of cancer biology. Currently, kinase inhibitors are among the key class of anticancer drugs.^32^ Among the potential synthetic lethal genes we identified, some encode kinases as shown in Table 6. Some clinically approved or experimentally active compounds that can inhibit these kinases are also presented in Table 6. These compounds may be effective in treating the cancers with mutations in synthetic lethal partners of the kinase genes.

**TABLE 6:**
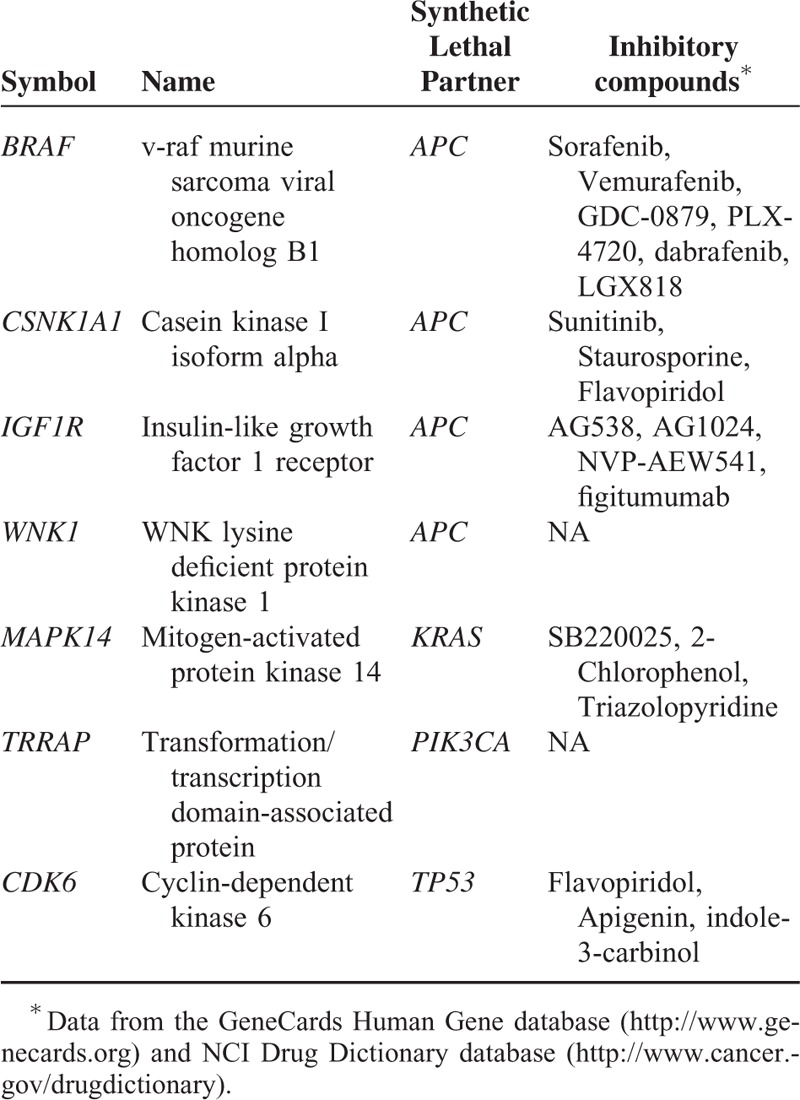
Potential Synthetic Lethal Kinase-Encoding Genes Identified

For some of the compounds whose GI50 values against NCI-60 cell lines are available in the National Cancer Institute's developmental therapeutics program (DTP) database website http://dtp.nci.nih.gov/dtpstandard/dwindex/index.jsp, we compared their drug sensitivity (GI50) between the cell lines with mutations in their synthetic lethal partners and the cell lines without such mutations using *t*-test statistics (1-sided, the hypothesis of higher sensitivity in the mutant cell lines). Although few of the drug sensitivity differences were statistically significant (the statistical power of the comparison was limited by the number of cell lines with mutations and compounds collected), the compound sunitinib targeting the kinase *CSNK1A1* shows higher sensitivity in the *APC* mutant cell lines than in the *APC* wide-type cell lines (*P* value = 0.0028), indicating that there exists a synthetic lethality relationship between *CSNK1A1* and *APC*.

### CDK4/6 Inhibitor PD-0332991 Selectively Inhibited Proliferation of TP53 Mutant Cells

**FIGURE 1:**
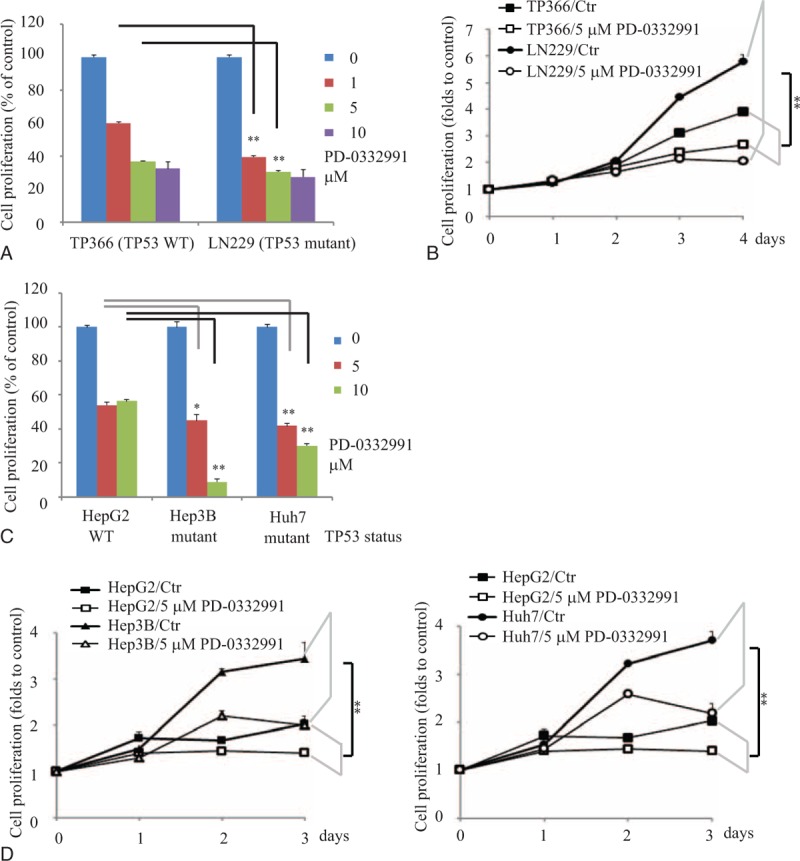
PD-0332991 selectively inhibited proliferation of *TP53* mutant cells. A, Glioblastoma TP366 and LN229 cells were treated with different concentrations of PD-033991 for 3 days followed by MTT assay. B, Glioblastoma TP366 and LN229 cells were treated with 5 μM PD-033991 for indicated time points followed by CCK8 assay. C, Hepatocellular carcinoma HepG2, Hep3B, and Huh7 cells were treated with indicated concentrations of PD-033991 for 3 days followed by MTT assay. D, Hepatocellular carcinoma HepG2, Hep3B, and Huh7 cells were treated with 5 μM PD-033991 for indicated time points followed by CCK8 assay. Data were expressed as mean ± S.E. ∗, *P* < 0.05; ∗∗, *P* < 0.01 (n = 4 or 6, the experiments were repeated at least twice).

In addition to several aforementioned computational evidences, to validate the synthetic lethal interaction between *TP53* and *CDK4/6*, we treated the *TP53* wild-type cells TP366, as well as *TP53* mutant cells LN229, with the inhibitor of *CDK4/6* PD-0332991. We found that PD-0332991 had less effect on the proliferation of TP366 cells with wild-type *TP53*, but induced greater proliferation reduction in LN229 cells with mutant *TP53* (Figure 1A and B). This indicates that synthetic lethal effects may exist between *TP53* and *CDK4/6*. Furthermore, we also checked hepatocellular carcinoma cell lines with or without *TP53* mutation with different concentrations of PD-0332991. PD-0332991 had stronger antiproliferation effects at Hep3B (*TP53* deficient) and Huh7 (*TP53* mutant) cells, compared with HepG2 with wild-type *TP53* (Figure 1C and D). In these experiments, we observed significant synthetic lethality between *TP53* and *CDK4/6*.

### Synthetic Lethal Interactions Between TDO2, CTNNB1, CSNK1A1, and APC

**FIGURE 2:**
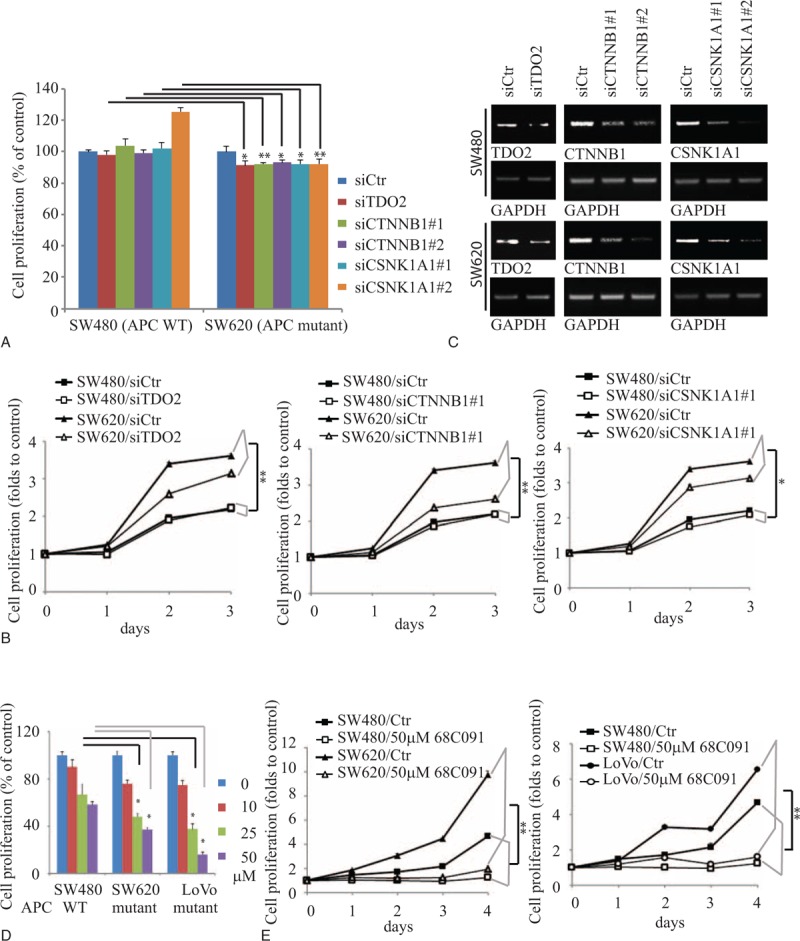
*TDO2*, *CTNNB1*, and *CSNK1A1* were validated as synthetic lethal to *APC*. A, B, Knockdown of *TDO2*, *CTNNB1*, or *CSNK1A1* inhibited the proliferation of *APC* mutant cells SW620, not *APC* wild-type cells SW480. SW480, and SW620 cells were transiently transfected with control siRNA or siTDO2, siCTNNB1, and siCSNK1A1 for 3 days or indicated time points followed by MTT assay or CCK8 assay. C, The siRNA knockdown effects were confirmed by RT-PCR. D, E, 680C91 selectively inhibited proliferation of *APC* mutant cells. Colorectal adenocarcinoma SW480, SW620, and LoVo cells were treated with indicated concentrations of 680C91 for 3 days followed by MTT assay (D) or treated with 50 μM 680C91 for indicated time points followed by CCK8 assay (E). Data were expressed as mean ± S.E. ∗, *P* < 0.05; ∗∗, *P* < 0.01 (n≥4, the experiments were repeated at least twice).

To validate the predicted *s*ynthetic lethal partners of *APC*, including *TDO2*, *CTNNB1*, and *CSNK1A1* in Table 2, we applied siRNA to knock down each of them at both *APC* wild-type cell SW480 and *APC* mutant cell SW620. Compared with the scrambled siRNA, 2 of the *CTNNB1*, *CSNK1A1*, and 1 of the *TDO2* siRNAs had significant enhanced inhibition on the proliferation of SW620 cells compared with SW480 cells (Figure 2A). We also monitored the growth curves of SW480 and SW620 after knocking down *TDO2*, *CTNNB1*, and *CSNK1A1*, respectively and observed that the *TDO2*, *CTNNB1*, and *CSNK1A1* siRNA strongly inhibit the growth of SW620 cells and slightly inhibit the growth of SW480 (Figure 2B). The siRNA knockdown efficiency was validated by RT-PCR (Figure 2C). To further check if there is a synthetic lethal interaction between *APC* and *TDO2*, *APC* wild-type cells SW480 and mutant cells SW620 and LoVo were treated with *TDO2* inhibitor 680C91. We observed that high concentrations of 680C91 (25–50 μM) led to significant decrease of cell proliferation for APC mutant cells SW620 and LoVo compared with APC wild-type cells SW480 (Figure 2D and E). These results suggest that there are synthetic lethal effects between *TDO2, CTNNB1, CSNK1A1*, and *APC*, respectively.

## DISCUSSION

In this study, we explored malignancy of cancers and synthetic lethal interactions relevant to the cancer driver genes based on publicly available gene mutation information in cancer cell lines and tumors with the computational approach. By comparisons of doubling time and MDR between the mutant and wild-type NCI-60 cell lines, we revealed that mutations of *APC*, *KRAS*, and *PIK3CA* might correlate with enhanced malignancy of cancer cells.

Ideally, anticancer drugs can selectively kill cancer cells but spare normal cells. Discovery of such drug targets is essential to develop effective anticancer drugs. Such targets are defined as cancer-specific vulnerabilities or as synthetic lethal interactions with cancer-related genetic mutations.^33^ The search for synthetic lethal partners for the genes that are frequently mutated in cancers but dodge drug inhibition has become a focus in cancer biology.

Based on the GPV concept, we identified a list of potential synthetic lethal genes to *APC*, *KRAS*, *BRAF*, *PIK3CA*, and *TP53* which are frequently mutated in cancers and are hardly druggable. Literature survey shows that some of the synthetic lethal relationships we predicted such as *TP53* and *TACC3*, *DOCK5* and *KRAS*, *RGS2* and *KRAS*, and *F2RL3* and *KRAS* have been experimentally verified to be authentic, indicating a certain rationality of our methods.

Our results show that the mean of *CTNNB1*'s GPVs in the *APC* mutant cell lines is 20% of that in the *APC* wild-type cell lines (*P* value <10^–7^), and *CTNNB1* has significantly higher level of expression in *APC* mutant cancer cell lines than in wide-type cell lines (*P* value = 0.015, fold-change = 1.52). These computational results strongly indicate that the *APC* mutant cell lines might depend on the *CTNNB1* gene for survival much more than the *APC* wild-type cell lines and therefore there may exist a synthetic lethal relationship between *CTNNB1* and *APC*. Indeed, our experiments verified the synthetic lethal relationship between them. *CTNNB1* has been shown to play an important role in regulation of cell adhesion and gene transcription, and its deregulation has been associated with many cancers.^34^ Thus, *CTNNB1* inhibitors are useful in treatment of many cancers including *APC* mutant cancers. Another gene *TDO2* has significant lower GPVs in the *APC* mutant cell lines than in wide-type cell lines (*P* value <10^–4^), and significantly higher level of expression in *APC* mutant cancer cell lines than in wide-type cell lines (*P* value = 0.021, fold-change = 3.02). Our experiments also verified the synthetic lethal relationship between *TDO2* and *APC* to be true. *TDO2* encodes a protein that may play a role in cancer through the suppression of antitumor immune responses.^35^ Thus, the associations of *APC*, *TDO2* and antitumor immune responses are worthy to be further investigated since cancer immunotherapy could be a fundamental breakthrough in cancer treatment.^36^*CDK6* has significant lower GPVs in the *TP53* mutant cell lines than in wide-type cell lines (*P* value <10^–4^), and significantly higher level of expression in *TP53* mutant cancer cell lines than in wide-type cell lines (*P* value <10^–7^, fold-change = 1.56). Again, our experiments confirmed the synthetic lethal relationship between *CDK6* and *TP53*.

In this study, we found that only a small portion of the potential synthetic lethal genes we computationally identified showed significantly higher expression in the mutant cell lines or tumors. There are several reasons to explain this result: first, sample sizes of the cell lines and the GBM tumors are relatively small; second, only 2 types of tumors were examined (if we examine many other tumor types, we may find much more of the potential synthetic lethal genes with higher expression in mutant than in wild-type tumors); third, the synthetic lethal interaction does not necessarily mean the expression difference between mutant and wild-type tumors. Here, we present the highly expressed genes in mutant cell lines or tumors to show that these genes are more likely to have synthetic lethal interactions with the cancer driver genes. However, we cannot exclude the synthetic lethal relationships for the genes which do not show significantly higher expression in the mutant cell lines or tumors relative to the wild-type counterparts. The synthetic lethal relationships need to be validated by experiments finally.

Furthermore, we selected some pairs of the synthetic lethal genes we computationally identified to perform experimental validation. Our experiments verified 4 genes to be truly synthetic lethal to the cancer driver genes including *CDK6* (with *TP53*), *TDO2*, *CTNNB1*, and *CSNK1A1* (with *APC*). Interestingly, 3 of them (*CDK6*, *TDO2*, and *CTNNB1*) show significantly higher expression in the tumors or cell lines with mutations of their synthetic lethal partners than in those without such mutations. In addition, 2 of them (*CDK6* and *CSNK1A1*) encode kinases for which small molecule inhibitors may be utilized to treat tumors with mutations of their synthetic lethal partners (*TP53* or *APC*).

The concept of RNAi synthetic lethality screening can be extended to drug synthetic lethality screening. It is known that single-agent targeted therapy is often in-efficacious or prune to relapse due to drug resistance caused by other genes’ mutations and bypassing the cell signaling pathway targeted by such single-agent at a system level. If we can find drug combinations that target these synthetic lethal partners simultaneously, we could repropose some drugs on personal precise medicine to obtain therapeutic solution to drug resistance.

## Supplementary Material

Supplemental Digital Content
