# Supplementary material for: Malignancy of Cancers and Synthetic Lethal Interactions Associated With Mutations of Cancer Driver Genes
Source: Medicine (Baltimore). 2016 Mar 3;95(8):e2697. doi: 10.1097/MD.0000000000002697 (PMC4778998; doi:10.1097/MD.0000000000002697)
Supplement: Supplemental Digital Content [file medi-95-e2697-s001.doc]

**Table S1.** NCI-60 cell lines’ phenotype data (doubling time, multidrug resistance, gene mutation status), 102 Achilles cancer cell lines’ phenotype data (gene mutation status and GPVs), and TCGA samples (gene mutation status)

**Gene mutant status in NCI-60 cell lines (data from http://www.sanger.ac.uk/genetics/CGP/NCI60/), and multidrug resistance (MDR) and doubling time phenotypes for NCI-60 cell lines (data from** [**http://discover.nci.nih.gov/cellminer/celllineMetadata.do**](http://discover.nci.nih.gov/cellminer/celllineMetadata.do)**)**

| Cell line | APC_mutant | BRAF_mutant | CDKN2A_mutant | KRAS_mutant | PIK3CA_mutant | PTEN_mutant | TP53_Mutatant | MDR | Doubling time |
| --- | --- | --- | --- | --- | --- | --- | --- | --- | --- |
| CCRF-CEM | no | no | yes | yes | no | yes | yes | 35 | 26.7 |
| UACC-62 | no | yes | yes | no | no | yes | no | 8 | 31.3 |
| 786-0 | no | no | yes | no | no | yes | yes | -44 | 22.4 |
| MOLT-4 | no | no | yes | no | no | yes | yes | 10 | 27.9 |
| RXF393 | no | no | yes | no | no | yes | yes | 42 | 62.9 |
| SF-295 | no | no | yes | no | no | yes | yes | 91 | 29.5 |
| SNB-19 | no | no | yes | no | no | yes | yes | -41 | 34.6 |
| U251 | no | no | yes | no | no | yes | yes | -19 | 23.8 |
| IGROV-1 | no | no | no | no | no | yes | yes | 20 | 31 |
| PC-3 | no | no | no | no | no | yes | yes | 11 | 27.1 |
| SF539 | no | no | no | no | no | yes | yes | -40 | 35.4 |
| KM12 | yes | no | no | no | no | yes | yes | -19 | 23.7 |
| HCT-116 | no | no | yes | yes | yes | no | no | 26 | 17.4 |
| NCI-H460 | no | no | yes | yes | yes | no | no | 25 | 17.8 |
| HCT-15 | yes | no | no | yes | yes | no | yes | 414 | 20.6 |
| MCF7 | no | no | yes | no | yes | no | no | 14 | 25.4 |
| SK-OV-3 | yes | no | yes | no | yes | no | yes | 15 | 48.7 |
| HT-29 | yes | yes | no | no | yes | no | yes | 15 | 19.5 |
| T47D | no | no | no | no | yes | no | yes | 19 | 45.5 |
| MDA-MB-231 | no | yes | yes | yes | no | no | yes | 29 | 41.9 |
| A549 | no | no | yes | yes | no | no | no | 10 | 22.9 |
| HOP62 | no | no | yes | yes | no | no | no | 61 | 39 |
| OVCAR-5 | no | no | yes | yes | no | no | no | 13 | 48.8 |
| NCI-H23 | no | no | no | yes | no | no | yes | -2 | 33.4 |
| RPMI-8226 | no | no | no | yes | no | no | yes | -3 | 33.5 |
| SW620 | yes | no | no | yes | no | no | yes | 31 | 20.4 |
| LOXIMVI | no | yes | yes | no | no | no | no | 12 | 20.5 |
| M14 | no | yes | yes | no | no | no | yes | 14 | 26.3 |
| MALME-3M | no | yes | yes | no | no | no | no | 22 | 46.2 |
| MDA-MB-435 | no | yes | yes | no | no | no | yes | 20 | 25.8 |
| SK-MEL-5 | no | yes | yes | no | no | no | no | 12 | 25.2 |
| A498 | no | no | yes | no | no | no | no | 78 | 66.8 |
| ACHN | no | no | yes | no | no | no | no | 120 | 27.5 |
| CAKI-1 | no | no | yes | no | no | no | no | 171 | 39 |
| DU145 | no | no | yes | no | no | no | yes | 4 | 32.3 |
| HL-60 | no | no | yes | no | no | no | yes | -11 | 28.6 |
| HOP-92 | no | no | yes | no | no | no | yes | -4 | 79.5 |
| K-562 | no | no | yes | no | no | no | yes | -1 | 19.6 |
| NCI-H226 | no | no | yes | no | no | no | no | 7 | 61 |
| SF-268 | no | no | yes | no | no | no | yes | -38 | 33.1 |
| SR | no | no | yes | no | no | no | no | 4 | 28.7 |
| UO-31 | no | no | yes | no | no | no | no | 59 | 41.7 |
| Hs-578-T | no | no | yes | no | no | no | no | NA | 53.8 |
| SK-MEL-28 | no | yes | no | no | no | no | yes | 11 | 35.1 |
| UACC-257 | no | yes | no | no | no | no | no | 26 | 38.5 |
| COLO-205 | yes | yes | no | no | no | no | yes | 7 | 23.8 |
| BT-549 | no | no | no | no | no | no | yes | -45 | 53.9 |
| EKVX | no | no | no | no | no | no | yes | -9 | 43.6 |
| NCI_ADR-RES | no | no | no | no | no | no | no | 352 | 34 |
| NCI-H322M | no | no | no | no | no | no | yes | -10 | 35.3 |
| NCI-H522 | no | no | no | no | no | no | yes | 16 | 38.2 |
| OVCAR-3 | no | no | no | no | no | no | yes | -12 | 34.7 |
| OVCAR-4 | no | no | no | no | no | no | yes | -4 | 41.4 |
| OVCAR-8 | no | no | no | no | no | no | no | 7 | 26.1 |
| SK-MEL-2 | no | no | no | no | no | no | yes | 14 | 45.5 |
| SN12C | no | no | no | no | no | no | yes | -86 | 29.5 |
| SNB-75 | no | no | no | no | no | no | yes | -38 | 62.8 |
| TK10 | no | no | no | no | no | no | yes | -4 | 51.3 |
| HCC2998 | yes | no | no | no | no | no | yes | -5 | 31.5 |

**Gene mutant status in 102 cancer cell lines (data from the website: http://www.broadinstitute.org/IGP/data/browseData?conversationPropagation=begin and** [**http://www.broadinstitute.org/ccle/data/browseData?conversationPropagation=begin**](http://www.broadinstitute.org/ccle/data/browseData?conversationPropagation=begin)**)**

| **Cancer cell lines** | **KRAS mutant status** | **PIK3CA mutant status** | **BRAF mutant status** | **APC mutant status** | **TP53 mutant status** |
| --- | --- | --- | --- | --- | --- |
| X786O_KIDNEY | NA | NA | NA | wildtype | wildtype |
| A204_SOFT_TISSUE | wildtype | wildtype | wildtype | wildtype | wildtype |
| A2058_SKIN | wildtype | wildtype | mutant | wildtype | mutant |
| A2780_OVARY | wildtype | wildtype | wildtype | wildtype | wildtype |
| A549_LUNG | mutant | wildtype | wildtype | wildtype | wildtype |
| AGS_STOMACH | mutant | mutant | wildtype | wildtype | wildtype |
| ASPC1_PANCREAS | mutant | wildtype | wildtype | wildtype | mutant |
| BXPC3_PANCREAS | wildtype | wildtype | wildtype | wildtype | mutant |
| C2BBE1_LARGE_INTESTINE | wildtype | wildtype | wildtype | mutant | mutant |
| CAOV3_OVARY | wildtype | wildtype | wildtype | wildtype | mutant |
| CAOV4_OVARY | wildtype | wildtype | wildtype | wildtype | mutant |
| CFPAC1_PANCREAS | mutant | wildtype | wildtype | wildtype | mutant |
| CH157MN_CENTRAL_NERVOUS_SYSTEM | NA | NA | NA | NA | wildtype |
| COLO205_LARGE_INTESTINE | wildtype | wildtype | mutant | mutant | wildtype |
| COLO704_OVARY | wildtype | wildtype | wildtype | wildtype | wildtype |
| COLO741_SKIN | wildtype | wildtype | mutant | NA | mutant |
| COV362_OVARY | NA | NA | NA | wildtype | mutant |
| COV434_OVARY | NA | NA | NA | NA | wildtype |
| COV504_OVARY | wildtype | wildtype | wildtype | wildtype | mutant |
| DLD1_LARGE_INTESTINE | mutant | mutant | wildtype | wildtype | wildtype |
| EFO21_OVARY | wildtype | wildtype | wildtype | wildtype | wildtype |
| EFO27_OVARY | wildtype | wildtype | wildtype | wildtype | mutant |
| F5_CENTRAL_NERVOUS_SYSTEM | NA | NA | NA | NA | wildtype |
| GP2D_LARGE_INTESTINE | mutant | mutant | wildtype | mutant | wildtype |
| HCC2814_LUNG | wildtype | NA | wildtype | NA | wildtype |
| HCC364_LUNG | wildtype | wildtype | mutant | NA | wildtype |
| HCC70_BREAST | wildtype | wildtype | wildtype | wildtype | mutant |
| HCC827_LUNG | wildtype | wildtype | wildtype | wildtype | mutant |
| HEC1A_ENDOMETRIUM | mutant | mutant | wildtype | wildtype | mutant |
| HEYA8_OVARY | mutant | wildtype | wildtype | wildtype | wildtype |
| HL60_HAEMATOPOIETIC_AND_LYMPHOID_TISSUE | wildtype | wildtype | wildtype | wildtype | wildtype |
| HLF_LIVER | wildtype | wildtype | NA | wildtype | mutant |
| HPAC_PANCREAS | mutant | wildtype | wildtype | wildtype | wildtype |
| HS944T_SKIN | wildtype | wildtype | wildtype | wildtype | wildtype |
| HT29_LARGE_INTESTINE | wildtype | mutant | mutant | mutant | wildtype |
| HT55_LARGE_INTESTINE | wildtype | wildtype | wildtype | mutant | wildtype |
| HUG1N_STOMACH | wildtype | NA | wildtype | wildtype | mutant |
| HUTU80_SMALL_INTESTINE | wildtype | wildtype | wildtype | wildtype | wildtype |
| IGR39_SKIN | wildtype | wildtype | mutant | wildtype | mutant |
| IGROV1_OVARY | wildtype | wildtype | wildtype | wildtype | mutant |
| IOMMLEE_CENTRAL_NERVOUS_SYSTEM | wildtype | NA | mutant | NA | wildtype |
| JHESOAD1_OESOPHAGUS | NA | NA | NA | NA | wildtype |
| JHOC5_OVARY | NA | NA | NA | NA | wildtype |
| KM12_LARGE_INTESTINE | wildtype | wildtype | wildtype | wildtype | mutant |
| KMS12BM_HAEMATOPOIETIC_AND_LYMPHOID_TISSUE | wildtype | wildtype | wildtype | wildtype | wildtype |
| KP1NL_PANCREAS | wildtype | wildtype | wildtype | wildtype | wildtype |
| KP4_PANCREAS | mutant | wildtype | wildtype | wildtype | wildtype |
| KURAMOCHI_OVARY | wildtype | wildtype | wildtype | wildtype | mutant |
| KYSE150_OESOPHAGUS | wildtype | wildtype | wildtype | wildtype | mutant |
| KYSE30_OESOPHAGUS | wildtype | wildtype | wildtype | wildtype | mutant |
| KYSE450_OESOPHAGUS | wildtype | wildtype | wildtype | wildtype | mutant |
| KYSE510_OESOPHAGUS | wildtype | mutant | wildtype | wildtype | wildtype |
| L33_PANCREAS | mutant | wildtype | wildtype | wildtype | wildtype |
| LN215_CENTRAL_NERVOUS_SYSTEM | NA | NA | NA | NA | wildtype |
| LN229_CENTRAL_NERVOUS_SYSTEM | wildtype | wildtype | wildtype | wildtype | mutant |
| LN319_CENTRAL_NERVOUS_SYSTEM | NA | NA | NA | NA | wildtype |
| LN464_CENTRAL_NERVOUS_SYSTEM | NA | NA | NA | NA | wildtype |
| LOVO_LARGE_INTESTINE | mutant | wildtype | wildtype | mutant | wildtype |
| LS411N_LARGE_INTESTINE | wildtype | wildtype | mutant | mutant | mutant |
| LS513_LARGE_INTESTINE | mutant | wildtype | wildtype | wildtype | wildtype |
| MDAMB453_BREAST | wildtype | mutant | wildtype | wildtype | wildtype |
| MIAPACA2_PANCREAS | mutant | wildtype | wildtype | wildtype | mutant |
| NCIH1650_LUNG | wildtype | wildtype | wildtype | wildtype | mutant |
| NCIH196_LUNG | wildtype | wildtype | wildtype | wildtype | mutant |
| NCIH1975_LUNG | wildtype | mutant | wildtype | wildtype | wildtype |
| NCIH2122_LUNG | mutant | wildtype | wildtype | wildtype | mutant |
| NCIH2171_LUNG | wildtype | wildtype | wildtype | wildtype | mutant |
| NCIH508_LARGE_INTESTINE | wildtype | mutant | mutant | wildtype | wildtype |
| NCIH661_LUNG | wildtype | wildtype | wildtype | wildtype | mutant |
| NCIH82_LUNG | wildtype | wildtype | wildtype | wildtype | wildtype |
| NIHOVCAR3_OVARY | wildtype | wildtype | wildtype | wildtype | mutant |
| OE33_OESOPHAGUS | wildtype | wildtype | wildtype | wildtype | mutant |
| OV90_OVARY | wildtype | wildtype | wildtype | wildtype | mutant |
| OVCAR4_OVARY | wildtype | wildtype | wildtype | wildtype | mutant |
| OVCAR8_OVARY | wildtype | wildtype | wildtype | wildtype | mutant |
| OVISE_OVARY | wildtype | mutant | wildtype | wildtype | wildtype |
| OVMANA_OVARY | wildtype | wildtype | wildtype | wildtype | wildtype |
| PANC0327_PANCREAS | mutant | wildtype | wildtype | wildtype | wildtype |
| PANC0813_PANCREAS | mutant | wildtype | wildtype | wildtype | wildtype |
| PANC1005_PANCREAS | mutant | wildtype | wildtype | wildtype | mutant |
| QGP1_PANCREAS | mutant | wildtype | wildtype | wildtype | wildtype |
| RKN_OVARY | mutant | wildtype | wildtype | wildtype | wildtype |
| RKO_LARGE_INTESTINE | wildtype | mutant | mutant | wildtype | wildtype |
| RMGI_OVARY | wildtype | wildtype | wildtype | wildtype | wildtype |
| RT112_URINARY_TRACT | wildtype | wildtype | wildtype | wildtype | mutant |
| SF767_CENTRAL_NERVOUS_SYSTEM | NA | NA | NA | NA | wildtype |
| SJSA1_BONE | wildtype | wildtype | wildtype | wildtype | wildtype |
| SKCO1_LARGE_INTESTINE | mutant | wildtype | wildtype | mutant | wildtype |
| SLR21_KIDNEY | NA | NA | NA | NA | wildtype |
| SNU840_OVARY | NA | NA | NA | NA | wildtype |
| SNUC1_LARGE_INTESTINE | wildtype | wildtype | wildtype | wildtype | wildtype |
| SNUC2A_LARGE_INTESTINE | mutant | wildtype | wildtype | wildtype | mutant |
| SU8686_PANCREAS | mutant | wildtype | wildtype | wildtype | mutant |
| SW480_LARGE_INTESTINE | mutant | wildtype | wildtype | wildtype | wildtype |
| SW48_LARGE_INTESTINE | wildtype | wildtype | wildtype | mutant | wildtype |
| TE15_OESOPHAGUS | wildtype | wildtype | wildtype | wildtype | wildtype |
| TE9_OESOPHAGUS | wildtype | wildtype | wildtype | wildtype | mutant |
| TOV112D_OVARY | wildtype | wildtype | wildtype | wildtype | wildtype |
| TOV21G_OVARY | mutant | mutant | wildtype | NA | wildtype |
| TT_OESOPHAGUS | wildtype | wildtype | wildtype | wildtype | mutant |
| TYKNU_OVARY | wildtype | wildtype | wildtype | wildtype | mutant |
| U251MG_CENTRAL_NERVOUS_SYSTEM | wildtype | wildtype | wildtype | wildtype | wildtype |

**Table S2**. Summary of the class comparisons of doubling time and MDR

| **Mutated Gene** | **Class** | **# Samples** ^a^ |
| --- | --- | --- |
| *APC* | *APC* mutant / wild-type | 59 (7/52) |
| *BRAF* | *BRAF* mutant / wild-type | 59 (11/48) |
| *CDKN2A* | *CDKN2A* mutant / wild-type | 59 (33/26) |
| *KRAS* | *KRAS* mutant / wild-type | 59 (11/48) |
| *PIK3CA* | *PIK3CA* mutant / wild-type | 59 (7/52) |
| *PTEN* | *PTEN* mutant / wild-type | 59 (12/47) |

^a^ The sample size of mutant and wide-type NCI-60 cell lines is given in parenthesis.

**Table S3**. Summary of the class comparisons of gene phenotype

| **Mutated Gene** | **Class** | **# Samples** ^b^ |
| --- | --- | --- |
| *APC* | *APC* mutant / wild-type | 86 (9/77) |
| *KRAS* | *KRAS* mutant / wild-type | 89 (25/64) |
| *BRAF* | *BRAF* mutant / wild-type | 88 (10/78) |
| *PIK3CA* | *PIK3CA* mutant / wild-type | 86 (12/74) |
| *TP53* | *TP53* mutant / wild-type | 102 (43/59) |

^b^ The sample size of mutant and wide-type Achilles cancer cell lines is given in parenthesis; the mutant status of some cell lines is unknown for the respective gene.

**Table S4.** Summary of the class comparisons of gene expression between mutant and wild-type TCGA tumors

| **Data set** ^c^ | **Class** | **# Samples** ^d^ |
| --- | --- | --- |
| GBM | *TP53* mutant / wild-type | 136 (47/89) |
| BRCA | *TP53* mutant / wild-type | 590 (201/389) |
|  | *PIK3CA* mutant / wild-type | 590 (194/396) |

^c^ GBM: glioblastoma multiforme; BRCA: breast invasive carcinoma.

^d^ The sample size of mutant and wide-type tumors is given in parenthesis.

**Table S5.** Results of statistical tests for each identified synthetic lethality gene (parametric pvalue, FDR, and permutation p-value)

| **Gene_Symbol** | **Parametric p-value** | **FDR** | **Permutation p-value** |
| --- | --- | --- | --- |
| CTNNB1 | < 1e-07 | < 1e-07 | < 1e-07 |
| CSNK1A1 | 0.0000002 | 0.000828 | < 1e-07 |
| BRAF | 0.0000087 | 0.0224 | 0.0002 |
| EIF3F | 0.0000352 | 0.0486 | 0.0001 |
| DHX9 | 0.0000549 | 0.0649 | 0.0001 |
| EIF2C3 | 0.0000631 | 0.0653 | < 1e-07 |
| GALNT8 | 0.0000808 | 0.0708 | < 1e-07 |
| TDO2 | 0.0000855 | 0.0708 | 0.0002 |
| HSPA8 | 0.0001046 | 0.0787 | < 1e-07 |
| EIF3L | 0.0001243 | 0.0858 | 0.0001 |
| IQGAP1 | 0.00015 | 0.0893 | 0.0002 |
| RING1 | 0.0001576 | 0.0893 | 0.0004 |
| MYCL2 | 0.0001618 | 0.0893 | 0.0001 |
| PNPLA2 | 0.0002021 | 0.099 | 0.0002 |
| EYA1 | 0.0002033 | 0.099 | 0.0002 |
| SEZ6L2 | 0.0002213 | 0.102 | 0.0002 |
| POLG2 | 0.0002708 | 0.112 | 0.0007 |
| DYNC1H1 | 0.0002811 | 0.112 | 0.0003 |
| PAPD5 | 0.0002853 | 0.112 | 0.0005 |
| RPS10 | 0.0003335 | 0.126 | 0.0007 |
| C1orf9 | 0.0003806 | 0.131 | 0.0002 |
| CXCR2 | 0.0003807 | 0.131 | 0.0007 |
| ZNF781 | 0.0004033 | 0.134 | 0.0011 |
| ABHD2 | 0.0005305 | 0.155 | 0.0007 |
| WNK1 | 0.0005382 | 0.155 | 0.0015 |
| IGF1R | 0.0005902 | 0.155 | 0.0007 |
| BCL2L1 | 0.0006086 | 0.155 | 0.0008 |
| EIF3FP3 | 0.0006621 | 0.155 | 0.0003 |
| ADSL | 0.0006624 | 0.155 | 0.0018 |
| OR1S2 | 0.0006807 | 0.155 | 0.0008 |
| SLC9A5 | 0.0006914 | 0.155 | 0.0011 |
| DNAJC17 | 0.0007338 | 0.155 | 0.0003 |
| XAB2 | 0.0007534 | 0.155 | 0.0012 |
| ANAPC4 | 0.0007684 | 0.155 | 0.0011 |
| NPR3 | 0.000784 | 0.155 | 0.0006 |
| BIRC5 | 0.0007964 | 0.155 | 0.0014 |
| EEF2 | 0.0008725 | 0.157 | 0.0011 |
| JUP | 0.0009029 | 0.157 | 0.0008 |
| PRSS42 | 0.0009101 | 0.157 | 0.001 |
| MED13 | 0.0009505 | 0.161 | 0.0007 |

| **Gene_Symbol** | **Parametric p-value** | **FDR** | **Permutation p-value** |
| --- | --- | --- | --- |
| BLMH | 0.0000327 | 0.061 | 0.0001 |
| DNAJB11 | 0.0000328 | 0.061 | < 1e-07 |
| CELF6 | 0.0000366 | 0.061 | 0.0001 |
| KIRREL3 | 0.0000442 | 0.061 | < 1e-07 |
| SLC7A11 | 0.0000643 | 0.063 | 0.0001 |
| HAVCR1 | 0.0000685 | 0.063 | 0.0001 |
| GPR34 | 0.0000965 | 0.0791 | 0.0001 |
| KCNJ6 | 0.0001082 | 0.0791 | < 1e-07 |
| CD3D | 0.0001147 | 0.0791 | 0.0001 |
| DOCK5 | 0.0001243 | 0.0792 | < 1e-07 |
| BRF2 | 0.0001456 | 0.0861 | < 1e-07 |
| GNPNAT1 | 0.0002507 | 0.138 | 0.0005 |
| TRMT5 | 0.0002881 | 0.149 | 0.0001 |
| GPR151 | 0.0004161 | 0.172 | 0.0005 |
| RHOV | 0.0005302 | 0.199 | 0.0004 |
| RGS2 | 0.0006064 | 0.203 | 0.0006 |
| MAPK14 | 0.0006121 | 0.203 | 0.001 |
| F2RL3 | 0.0006645 | 0.205 | 0.0006 |
| OR10A5 | 0.000667 | 0.205 | 0.0009 |
| MYCBP2 | 0.0008649 | 0.239 | 0.0003 |
| HNF1A | 0.0009009 | 0.24 | 0.0011 |

| **Gene_Symbol** | **Parametric p-value** | **FDR** | **Permutation p-value** |
| --- | --- | --- | --- |
| PCDHGB4 | 0.0005089 | 0.994 | 0.0004 |
| ZNF138 | 0.000577 | 0.994 | 0.0005 |
| CXCR2 | 0.0006001 | 0.994 | 0.0007 |
| CDH2 | 0.0008121 | 0.997 | 0.0005 |
| DGKA | 0.0008434 | 0.997 | 0.0013 |
|  |  |  |  |

| **Gene_Symbol** | **Parametric p-value** | **FDR** | **Permutation p-value** |
| --- | --- | --- | --- |
| DARS | 0.0000156 | 0.0431 | < 1e-07 |
| OGDH | 0.0000213 | 0.0441 | 0.0001 |
| KIAA1012 | 0.0000295 | 0.0489 | < 1e-07 |
| TRRAP | 0.0000482 | 0.054 | < 1e-07 |
| RBM17 | 0.0000529 | 0.054 | < 1e-07 |
| SLC25A3 | 0.0000587 | 0.054 | < 1e-07 |
| RPS17 | 0.0000745 | 0.0572 | < 1e-07 |
| PRPF8 | 0.000076 | 0.0572 | 0.0004 |
| ABCB7 | 0.0000901 | 0.0622 | 0.0002 |
| NUP93 | 0.0001176 | 0.0706 | 0.0002 |
| PRIM2 | 0.0001354 | 0.0747 | < 1e-07 |
| HNRNPA3 | 0.0002018 | 0.102 | 0.0002 |
| CACNA2D4 | 0.0002165 | 0.102 | 0.0001 |
| TUBG1 | 0.0002356 | 0.102 | 0.0007 |
| PRPF31 | 0.0002469 | 0.102 | 0.0004 |
| COPS6 | 0.0003297 | 0.118 | 0.0005 |
| PSMA1 | 0.0003547 | 0.118 | 0.0006 |
| SRRT | 0.0003571 | 0.118 | 0.0001 |
| PUF60 | 0.0004213 | 0.131 | 0.0006 |
| WBP11 | 0.0004281 | 0.131 | 0.0003 |
| C12orf24 | 0.0004862 | 0.133 | 0.0004 |
| EIF2S2 | 0.0004938 | 0.133 | 0.0006 |
| SNAPC3 | 0.0005277 | 0.133 | 0.0004 |
| BCS1L | 0.0005279 | 0.133 | 0.0007 |
| SLIT3 | 0.0005383 | 0.133 | 0.0008 |
| CDC5L | 0.0005472 | 0.133 | 0.001 |
| DUT | 0.0005478 | 0.133 | 0.0003 |
| RPS4X | 0.000573 | 0.136 | 0.0011 |
| AQR | 0.0006384 | 0.136 | 0.0013 |
| DLST | 0.0006466 | 0.136 | 0.001 |
| CWC15 | 0.0006587 | 0.136 | 0.0005 |
| PSMC4 | 0.0007152 | 0.137 | 0.0005 |
| PSMD4 | 0.0007332 | 0.137 | 0.001 |
| GPLD1 | 0.0007359 | 0.137 | 0.0006 |
| CASP8AP2 | 0.0007395 | 0.137 | 0.0011 |
| PIGL | 0.0007594 | 0.137 | 0.0013 |
| TSG101 | 0.0007862 | 0.137 | 0.0005 |
| CCT5 | 0.00079 | 0.137 | 0.0013 |
| YME1L1 | 0.0008089 | 0.137 | 0.0009 |
| PSMB2 | 0.0008099 | 0.137 | 0.0015 |
| SFRS7 | 0.0008946 | 0.146 | 0.001 |
| DLD | 0.0009213 | 0.146 | 0.0007 |
| DMRTA2 | 0.0009321 | 0.146 | 0.0013 |

| **Gene_Symbol** | **Parametric p-value** | **FDR** | **Permutation p-value** |
| --- | --- | --- | --- |
| CDK6 | 0.0000877 | 0.182 | 0.0001 |
| IFT57 | 0.00013 | 0.189 | 0.0001 |
| PRB1 | 0.0001594 | 0.189 | 0.0002 |
| OGDH | 0.0001595 | 0.189 | 0.0002 |
| POLR2E | 0.0003187 | 0.211 | 0.0002 |
| BCS1L | 0.0003276 | 0.211 | 0.0001 |
| ZNF691 | 0.0003613 | 0.211 | 0.0004 |
| SATB2 | 0.0003884 | 0.211 | 0.0003 |
| ETFB | 0.0004011 | 0.211 | 0.0001 |
| RRN3P1 | 0.0004078 | 0.211 | 0.0002 |
| LHX5 | 0.0005196 | 0.226 | 0.0013 |
| PRDM12 | 0.0007117 | 0.272 | 0.0009 |
| RBM17 | 0.0007738 | 0.272 | 0.0005 |
| CCT6A | 0.0008457 | 0.272 | 0.0008 |
| TACC3 | 0.0009067 | 0.272 | 0.0005 |
| MYC | 0.0009203 | 0.272 | 0.0006 |
| RPS4X | 0.0009477 | 0.272 | 0.0006 |
| NPAS2 | 0.0009851 | 0.272 | 0.0008 |
